# Comparison of Static Balance and the Role of Vision in Elite Athletes

**DOI:** 10.2478/hukin-2014-0030

**Published:** 2014-07-08

**Authors:** Raouf Hammami, David G Behm, Mokhtar Chtara, Aymen Ben Othman, Anis Chaouachi

**Affiliations:** 1 Tunisian Research Laboratory “Sport Performance Optimisation”, National Center of Medicine and Science in Sports, Tunis, Tunisia.; 2 School of Human Kinetics and Recreation, Memorial University of Newfoundland, St. John’s Newfoundland, Canada.

**Keywords:** Athletes, surface, vision, static balance

## Abstract

When prescribing balance exercises to athletes in different sports, it may be important to recognize performance variations. Indeed, how athletes from different sports perform on balance tests is not well understood. The goal of the present study was to compare static balance and the role of vision among elite sprinters, jumpers and rugby players. The modified clinical test of sensory interaction on balance (mCTSIB) was used to assess the velocity of the center-of-pressure (CoP) on a force platform during a 30 s bipedal quiet standing posture in 4 conditions: firm surface with opened and closed eyes, foam surface with opened and closed eyes. Three-factor ANOVA indicated a significant main effect for groups (F=21.69, df=2, p<0.001, η^2^ = 0.34). Significant main effect of vision (F=43.20, df=1, p<0.001, η^2^ = 0.34) and surface (F=193.41, df=1, p<0.001, η^2^ = 0.70) as well as an interaction between vision (eyes open, eyes closed) and surface (firm and foam) (F=21.79, df=1, p=0.001) were reported in all groups. The subsequent Bonferroni-Dunn post hoc test indicated that rugby players displayed better static balance than sprinters and jumpers (p=0.001). The comparison of sprinters and jumpers did not reveal significant differences (p>0.05). The nature of the sport practiced and the absence of visual control are linked to modify static balance in elite athletes. Coaches and strength and conditioning professionals are recommended to use a variety of exercises to improve balance, including both exercises with opened and closed eyes on progressively challenging surfaces in order to make decisions about tasks and sensory availability during assessment and training.

## Introduction

The ability to minimize postural sway has been defined as postural performance (Paillard et al., 2002). Balance has been shown to play a fundamental role in many athletic activities as well as sport specific postural control and may contribute to a successful performance although the relationship between balance ability and athletic performance is less clear (Alderton et al., 2003; Hryssomalis et al., 2011). Factors that alter postural control responses include sensory information obtained from the somatosensory, visual, and vestibular systems and motor responses that affect the quality and safety of performance during routine functional movements, athletic performance (Haryssomalis et al., 2011), coordination, joint range of motion (ROM), incremental exercise to fatigue (Erkmen et al., 2012) and strength (Grigg, 1994; Palmeiri, 2002). In order to have optimal balance, it is necessary that the three afferent systems of proprioception, vision and vestibular provide the necessary information for this performance.

Vision is responsible for the head’s position and movement in relation to the surrounding objects. Information acquired via vision is important for maintaining balance (Berthoz et al., 2001). Numerous studies have analyzed various motor control and orientation in space strategies by examining the coordination of the movement of the eyes, head, body, and limbs during a locomotor task (Cremieux, 1994; Imai, 2001; Paillard, 2006). However, controversy exists as to whether high level athletes demonstrate different postural control strategies (eyes open or closed and single- or double-leg stance) compared with others competing in different sports. Indeed, there is a lack of research evidence regarding whether balance assessment with eyes closed is more efficient than tests with opened eyes. Paillard and colleagues (2002) showed that judo athletes at the highest level of competition were more dependent on visual information to control their posture. Moreover, experienced athletes generally use specific sensory information in organizing posture in relation with the requirements of each discipline (Perrin, 1998; Vuillerme, 2001). For example, rugby players must control their posture in order to execute a variety of skills (e.g. high speed sprints and changes of direction as well as kicking the ball for passing or shooting). While performing these skills rugby players must also incorporate visual information about other team members and opponents (Brault et al., 2010). Hence, rugby training necessitates strong visual dependence in relation to the ball, opposing players and other team members. Based on the specificity of practice hypothesis, visual dependency during the execution of a motion depends on the individual, environment and task (Perrin et al., 2002). Similarly, Romero-Franco and colleagues (2012) studied the effect of a proprioceptive training program on center of gravity control in sprinters and stated that improvement in balance was reported when exercises were performed with eyes opened. This dependency on vision has been documented in other sports such as surfing (Chapman et al., 2008) and soccer (Burfield and Fischman, 1990). It was demonstrated that surfers had better results at anterior-posterior balance when compared with untrained subjects. Thereafter, surfers were able to partly transfer their static postural control function and were able to develop specific balance models (Chapman et al., 2008). In contrast, in individual sports such as judo and triathlon, athletes have been shown to have less dependency on vision (Williams, 2002; Nagy, 2004; Simonson, 2005).

Since dependence on vision is related to the nature of the sport, clarifying the influence of the type of activity and the importance of vision on changes in balance performance appears to be important. Hence, we expected that visual dependency, skills and sport activity may be related to balance performance in high-level athletes. The objective of this study was then to compare static balance and the role of vision in elite sprinters, jumpers and rugby players.

## Material and Methods

### Subjects

Twenty-four elite male athletes including sprinters, jumpers, and rugby players, members of the National athletics and Rugby teams agreed to participate in this study. The 3 groups of sprinters (21.83 ± 2.72 years; 71.87 ± 7.94 kg and 179.75 ± 5.25 cm), jumpers (22.91 ± 2.06 years; 73.37 ± 7.72 kg and 180.50 ± 5.6 cm) and rugby players (22.91 ± 2.06 years; 89.25 ± 3.19 kg and 180.87 ± 2.94 cm) consisted of 8 athletes each. None had a history of musculoskeletal, neurological, visual or orthopedic disorder that might affect their ability to perform balance tests. Written informed consent was obtained from all players after verbal and written explanation of the experimental design and potential risks of the study. The research was conducted according to the Declaration of Helsinki and the protocol fully approved by the Ethics Committee of the National Center of Medicine and Science in Sports of Tunis before the commencement of the study. All athletes were informed that they could withdraw from the study at any time without any consequences.

### Procedures

During the familiarization session prior to testing, subjects were instructed on the administration and recording of the tests as well as interview techniques. They were also instructed regarding the correct use and permitted to practice with the Neuro Com Balance Master ® platform. NeuroCom Balance Master ® is a computerized sensitive posturography device that measures athlete’s responses to the movement of a platform on which the subject is standing or sitting, then provides computer generated assessments of the athlete’s postural alignment and stability. Test circumstances (e.g., room illumination, temperature, noise) were in accordance with recommendations for posturographic testing (Shumway-Cook et al., 2000). Following the medical history interview, vision (with and without corrective lenses, in poor light) and history of dizziness evaluations, subjects performed a Modified Clinical Test for Sensory Interaction on Balance (mCTSIB). The mCTSIB was conducted on the Neuro Com Balance Master ® with a sampling frequency of 100 Hz, which examines postural sway during the 4 conditions assessed for the mCTSIB: “standing on a firm surface with eyes open”, “standing on a firm surface with eyes closed”, “standing on a foam surface with eyes open”, and “standing on a foam surface with eyes closed”. Composite sway was the mean sway speed averaged over the 4 conditions. Each condition was tested 3 times with a 1 min rest period between following trials. The order of completion of the 4 conditions was randomized for all participants.

Subjects stood straight and still on a force platform during three 30 s trials in each of the 4 conditions. For each condition, the subject’s feet were placed in the standard position recommended by the manufacturer of the Balance Master ®. Foot position was monitored throughout the test. If foot placement changed, the feet were again placed in the correct position. The mCTSIB gives 1 set of data collected by the computer from the 4 conditions. Data include mean center of pressure sway speed (which is measured in degrees per second over 30 s) and were collected with Neuro Com Balance Master ®. Software 3.4. We used center-of-pressure (CoP) speed for the 4 conditions and composite sway for statistical analysis. If the speed of CoP was low the subject was considered stable (Winter et al., 1995).

### Statistical analysis

Means ± standard deviations [SD] were used to describe variables. Before using parametric tests, the assumption of normality was verified using the Kolmogorov-Smirnov test. The data were analyzed using three-factor ANOVA (2×2×3). The within-subjects factors were 1) vision (eyes opened, eyes closed) and 2) the surface (firm, foam), with the between-subjects factors being the three groups (sprinters, jumpers, and rugby players). If significant main effects or interactions were present, a Bonferroni (Dunn) procedure was conducted. The effect size was calculated for all ANOVAs with the use of a partial eta-squared. Values of 0.01, 0.06 and above 0.15 were considered as small, medium and large, respectively (Cohen, 1988). To provide meaningful analysis for comparisons from small groups, the Cohen’s effect sizes (ES) between conditions were also calculated (Cohen, 1988) (small < 0.50, moderate = 0.50–0.79 and large > 0.80). Statistical analysis was performed using the SPSS software statistical package (SPSS Inc., Chicago, IL, version. 16.0), and statistical significance was set at p < 0.05.

## Results

Results are summarized in Table 1. For static balance (mCTSIB), which had a normal distribution, the three-factor ANOVA indicated a significant main effect of groups (F=21.69, df=2, p<0.001, η^2^ = 0.34). The subsequent Bonferroni-Dunn post hoc test indicated that the speed of the CoP was lower for rugby players than for sprinters and jumpers. Rugby players displayed better static balance (p=0.001) than sprinters (ES = 1.02) and jumpers (ES = 1.06).

The comparison of sprinters and jumpers did not reveal significant differences (p>0.05) (Table 2). The analysis revealed significant main effect of vision (F=43.20, df=1, p<0.001, η^2^ = 0.34). Static balance was better when athletes performed the test with open eyes than with eyes closed (p<0.001, ES = 0.90). There was no interaction between vision and the three groups (F=1.29, df=2, p>0.05, η^2^ = 0.03). Significant main effect was also observed in surface (F=193.41, df=1, p<0.001, η^2^ = 0.70). Static balance was better when athletes performed tasks on the firm surface than on the foam surface (p<0.001, ES = 3.09). There was an interaction between surface (firm and foam) and three groups (F=6.363, df=2, p=0.002, η^2^ = 0.0.14). Furthermore, there was an interaction between vision (eyes open, eyes closed) and surface (firm and foam) (F=21.79, df=1, p=0.001). No interaction between vision versus surface and groups was observed (F=1.94, df=2, p>0.05).

## Discussion

Some research suggests that superior performance among experienced athletes is largely the result of repetitive training experiences that influence motor responses and not the greater sensitivity of the vestibular system (Balter et al., 2004). Others argue that superior balance and performance is the result of training experiences that influence a person’s ability to attend to relevant proprioceptive and visual cues (Ashton et al., 2001). Although the idea that sport involvement improves balance is not new, our study extends this knowledge to particular sports and suggests that specific sensorimotor challenges, rather than just general sport activity, are important for the development of optimal balance.

Sprinters and jumpers demonstrated inferior static balance compared with rugby players. Rugby is a contact sport that necessitates an internal control of equilibrium when attempting to maintain balance while being hit by opponents (Brault et al., 2010). Rugby players must efficiently integrate internal (proprioception and vestibular systems) and external (visual) cues to achieve success (Brault et al., 2010). In contrast to jumpers and sprinters, rugby players must be able to maintain balance despite sudden changes of direction and impacts. In order to achieve it, rugby players would need to lower their centre of gravity, which requires a body reorientation strategy, that could decrease their balance potential (Sayers et al., 1999). In duels such as an attacker vs. a defender in rugby, successful body orientation/reorientation strategies are essential for successful balance performance (Brault et al., 2010). Similarly, players should be able to analyze game situations and select correct options, think ahead, predict with certainty what will happen from set attacking and defensive plays. They need to continuously process information very rapidly and combine several highly skilled tasks in order to bring about the desired outcome while maintaining balance (Wismentel et al., 2002).

So, practicing rugby seems to elicit long-term improvements in postural control. Additionally, recent studies have reported that elite athletes showed a greater “neural efficiency” than non-athletes, as a result of the practice of these sports (Filingeri et al., 2012; Juras et al., 2013). Paillard and colleagues (2002) did not observe any significant difference between the postural performances of two groups of judo athletes at different levels of competition when testing subjects with a classical bipedal standing task. These authors showed that judo athletes at the highest level of competition were more dependent on visual information to control their posture. Sirmen and colleagues (2008) compared the values of dynamic and static balance of karate and water polo athletes. Karate athletes were found to have a better balance pattern towards left backwards when compared with water polo athletes.

Furthermore, the absence of vision significantly disturbed postural control for all groups of athletes, as observed previously with many other sports such as shooting, judo, ballet dancing, and gymnastics (Perrin, 2002; Paillard, 2002). Our results showed that closing eyes increased postural sway in all groups of athletes. This finding could be explained by the fact that postural regulation developed in terms of visual control with sport training is not always transferable to upright stance situations (Asseman et al., 2004). Vuillerme and Nougier (2004) conducted tests on athletes from a variety of sports (e.g. gymnastics, football, handball) while they were tested with three different postural balance tests; one foot, both feet and one-foot on the mat. There were no significant differences between groups with eyes opened. However, it was observed that gymnasts demonstrated better results during eyes closed postural balance measurements. In this context, Smith and colleagues (2012) stated that exercises such as standing on unstable surfaces with eyes open instead of eyes closed and head back were more beneficial to the children’s postural stability control system. In contrast, it was demonstrated that higher visual dependency increases balance control perturbation during cognitive task fulfillment with high-level athletes (Slobounov et al., 2006). Romero-Franco and colleagues (2012) studied the effect of a proprioceptive training program on center of gravity control in sprinters and stated that improvement in balance was accomplished when exercises were performed with eyes opened. This statement is consistent with our results with all subjects relying on vision for maintaining their balance. For postural regulation, the reliance on visual cues increases for all groups demonstrating the role of vision for maintaining better balance in athletes. These findings are consistent with the literature (Perrin, 2002; Simmons, 2005; Williams, 2002; Harringe, 2008). Their studies found that if during task performance the visual cues are deleted, motor control would be at risk. Thereafter the loss of visual cues, will significantly affect motor control. Hallemans and colleagues (2009) found significant differences in sway with eyes opened and closed and stated that visual deprivation affects locomotion in both adults and children. These observations support the hypothesis that gait adaptations in situations of visual deprivation are related to balance problems. There seems to be a unique set of visual skills that are common to athletes in certain sports. In addition, visual performance measures vary between sports at the Olympic level. The ability to identify the visual needs for an athlete who wishes to participate in a given sport, and to correct any deficits an athlete may have, could lead to more success, at both elite and amateur levels (Portal et al., 1998). So, for maintaining good balance, it is necessary to include specific workouts containing exercises practiced with eyes opened and closed to restore specific motor skills likely to correct postural adjustments and to make decisions about tasks and sensory availability during assessment and training. However, with regard to the present study, it could be stated that a longer time acquisition than 30 seconds and a greater number of participants may have provided greater significant interaction results. The present duration and population sample size may have limited our ability to interpret and apply the results to a greater population.

## Conclusion

Our data show that sprinters, jumpers and rugby players differ in terms of static balance. Rugby players displayed better static balance compared with sprinters and jumpers. The nature of the sport practiced and the absence of visual control are linked to modify static balance in elite athletes.

For practical purposes, the results of this study will be of benefit to the practitioners seeking to identify postural profile and the importance of vision in sport performance. Coaches and strength and conditioning professionals are recommended to use a variety of exercises to improve balance, including both exercises with opened and closed eyes on progressively challenging surfaces in order to make decisions about tasks and sensory availability during assessment and training. This finding is important to make an appropriate adjustment for balance performance enhancement, in addition to traditional metabolic conditioning. It allows us to better test and monitor high level athletes requiring different skills and to evaluate the possible effects of training in elite athletes. Similar studies should be conducted on youth athletes.

## Figures and Tables

**Table 1:** Anthropometric characteristics of elite sprinters (n=8), jumpers (n=8), and rugby players (n=8)

*Groups*	*Age (years)*	*Body mass (kg)*	*Body height (cm)*
Sprinters	21.83 ± 2.72	71.87 ±7.94	179.75 ± 5.25
Jumpers	22.91±2.06	73.37± 7.72	180.50 ±5.6
Rugby players	22.91±2.06	89.25± 3.19^*^	180.87± 2.94

**Table 2:** Mean ±SD and 95% confidence intervals of the modified clinical test of sensory interaction on balance (mCTSIB) in sprinters, jumpers and rugby players

Groups	Vision	Surface	Mean ± SD (m·s^−1^)	95% Confidence Interval	
				Lower Bound	Upper Bound
Sprinters	Opened Eyes	Firm Surface	0.36 ± 0.17	.21	.51
		Foam Surface	0.81 ± 0.28	.66	.96

	Closed Eyes	Firm Surface	0.40 ± 0.14	.25	.55
		Foam Surface	1.50 ± 0.15	1.35	1.65

	Total	Opened Eyes	0.59 ± 0.32	.48	.69
		Closed Eyes	0.95 ± 0.59	.84	1.06

		Firm Surface	0.38 ± 0.15	.28	.49
		Foam Surface	1.16 ± 0.42	1.05	1.26

Jumpers	Opened Eyes	Firm Surface	0.27 ± 0.19	.12	.43
		Foam Surface	0.78 ± 0.38	.62	.93

	Closed Eyes	Firm Surface	0.43 ± 0.33	.27	.58
		Foam Surface	1.24 ± 0.26	1.09	1.39

	Total	Opened Eyes	0.53 ± 0.39	.42	.63
		Closed Eyes	0.83 ± 0.51	.73	.94

		Firm Surface	0.35 ± 0.27	.24	.46
		Foam Surface	1.01 ± 0.39	.90	1.11

Rugby players	Opened Eyes	Firm Surface	0.20 ± 0.11	.05	.35
		Foam Surface	0.46 ± 0.12	.31	.61

	Closed Eyes	Firm Surface	0.26 ± 0.09	.11	.41
		Foam Surface	0.79 ± 0.11	.64	.94

	Total	Opened Eyes	0.33 ± 0.17	.23	.44
		Closed Eyes	0.52 ± 0.29	.42	.63

		Firm Surface	0.23 ± 0.10	.13	.34
		Foam Surface	0.62 ± 0.20	.52	.73

All athletes	Opened Eyes	Firm Surface	0.28 ± 0.17	.19	.37
		Foam Surface	0.68 ± 0.31	.60	.77

	Closed Eyes	Firm Surface	0.36 ± 0.22	.28	.45
		Foam Surface	1.17 ± 0.35	1.09	1.26

	Total	Opened Eyes	0.48 ± 0.32	.42	.54
		Closed Eyes	0.77 ± 0.50	.71	.83

		Firm Surface	0.32 ± 0.20	.26	.38
		Foam Surface	0.93 ± 0.41	.87	.99

**Table 3:** The main effect of vision, surface, groups and interaction on balance.

Source		Mean Estimate	F	Sig.	Partial Eta Squared
Groups	Sprinters	0.77	21.69	**.001**	.34
	Jumpers	0.68			
	Rugby players	*0.43^a^*			
Vision	Opened Eyes	*0.48^b^*	43.20	**.001**	.34
	Closed Eyes	0.77			
Surface	Firm Surface	*0.32^c^*	193.41	**.001**	.70
	Foam Surface	0.93			
Groups * Vision			1.29	.282	.03
Groups * Surface			6.63	**.002**	.14
Vision* Surface			21.79	**.001**	.21
Groups * Vision * Surface			1.94	.151	.04

a = significant better static balance than sprinters and jumpers;

b= better static balance with opened eyes.

c = better static balance with firm surface.

## References

[b1-jhk-41-33] Alderton AK, Moritz U, Moe-Nilssen R (2003). Force plate and accelerometer measures for evaluating the effect of muscle fatigue on postural control during one legged stance. Physiother Res Int.

[b2-jhk-41-33] Ashton-Miller JA, Wojtus EM, Huston LJ, Fry-Welch D (2001). Can proprioception really be improved by exercises?. Knee Surg Sports Traumatol Arthrosc.

[b3-jhk-41-33] Asseman F, Caron O, Cremieux J (2004). Is there a transfer of postural ability from specific to unspecific postures in elite gymnasts?. Neurosci Lett.

[b4-jhk-41-33] Balter SGT, Stokroos RJ, Akkermans E, Kingma H (2004). Habituation to galvanic vestibular stimulation for analysis of postural control abilities in gymnasts. Neurosci Lett.

[b5-jhk-41-33] Berthoz A (2001). Neural basis of spatial orientation and memory of routes: topokinetic memory or topokinesthesic memory. Rev Neurol (Paris).

[b6-jhk-41-33] Brault S, Bideau B, Craig C, Kulp R (2010). Balancing deceit and disguise: How to successfully fool the defender in a 1 vs. 1 situation in rugby. Hum Mov Sci.

[b7-jhk-41-33] Burfield B, Fischman M (1990). Control of a ground-level ball as a function of skill level and sight of the foot. J Hum Mov Study.

[b8-jhk-41-33] Costill D, Maglischo E, Richardson A (1992). Swimming.

[b9-jhk-41-33] Chapman DW, Needham KJ, Allison GT, Lay B, Edwards DJ (2008). Effects of experience within a dynamic environment on postural control. Br J Sports Med.

[b10-jhk-41-33] Cohen J (1988). Statistical Power Analysis for the Behavioral Sciences.

[b11-jhk-41-33] Cremieux J, Mesure S (1994). Differential sensivity to static visual cues in the control of postural equilibrium in man. Percept Mot Skills.

[b12-jhk-41-33] Erkmen N, Suveren S, Göktepe AS (2012). Effects of Exercise Continued Until Anaerobic Threshold on Balance Performance in Male Basketball Players. J Hum Kinet.

[b13-jhk-41-33] Filingeri BA, Zangla D, Paoli A, Palma A (2012). Is karate effective in improving postural control?. Sci Mart Art.

[b14-jhk-41-33] Grigg P (1994). Peripheral neural mechanisms in proprioception. Sport Rehab.

[b15-jhk-41-33] Hallemans A, Beccu S, Van Loock K, Ortibus E, Truijen S, Aerts P (2009). Visual deprivation leads to gait adaptations that are age- and context-specific: I. Step-time parameters. Gait Posture.

[b16-jhk-41-33] Harringe ML, Halvorsen K, Renstrom P, Werner S (2008). Postural control measured as the center of pressure excursion in young female gymnasts with low back pain or lower extremity injury. Gait Posture.

[b17-jhk-41-33] Hrysomallis C (2011). Balance ability and athletic performance. Sports Med.

[b18-jhk-41-33] Imai T, Moore ST, Raphan T, Cohen B (2001). Interaction of the body, head and eyes during walking and turning. Exp Brain Res.

[b19-jhk-41-33] Juras G, Słomka K (2013). Anticipatory Postural Adjustments in Dart Throwing. J Hum Kinet.

[b20-jhk-41-33] Nagy E, Toth K, Janositz G, Kovacs G, Faherkiss A, Angyan L, Horvath G (2004). Postural control in athletes participating in an ironman triathlon. Eur J Appl Physiol.

[b21-jhk-41-33] Paillard T, Costes Salon C, Lafont C, Dupui P (2002). Are there differences in postural regulation according to the level of competition in judoists?. Br J Sports Med.

[b22-jhk-41-33] Paillard T, Noe F, Riviere T, Marion V, Montoya R, Dupui P (2006). Postural performance and strategy in the unipedal stance of soccer players at different levels of competition. J Athl Train.

[b23-jhk-41-33] Palmieri RM, Ingersoll CD, Stone MB, Krause BA (2002). Center-of-pressure Parameters used in the assessment of postural control. J Sport Rehabil.

[b24-jhk-41-33] Perrin P, Schneider D, Deviterne D, Perrot C, Constantinescu L (1998). Training improves the adaptation to changing visual conditions in maintaining human posture control in a test of sinusoidal oscillation of the support. Neurosci Lett.

[b25-jhk-41-33] Perrin PH, Deviterne D, Hugel F, Perrot C (2002). Judo better than dance develops sensorimotor adaptabilities involved in balance control. Gait Posture.

[b26-jhk-41-33] Portal JM, Romano PE (1998). Major review: ocular sighting dominance: a review and a study of athletic proficiency and eye-hand dominance in a collegiate baseball team. Binocul Vis Strabismus Q.

[b27-jhk-41-33] Romero-Franco N, Martínez-López E, Lomas-Vega R, Hita-Contreras F, Martínez-Amat A (2012). Effects of proprioceptive training program on core stability and center of gravity control in sprinters. J Strength Cond Res.

[b28-jhk-41-33] Sayers M (1999). Sagittal plane running kinematics of elite’s rugby players. J Appl Biomech.

[b29-jhk-41-33] Shumway-Cook A, Brauer S, Woollacott M (2000). Predicting the probability for falls in community-dwelling older adults using the Timed Up & Go Test. Phys Ther.

[b30-jhk-41-33] Simmons RW (2005). Sensory organization determinates of postural stability in trained ballet dancers. Int J Neurosci.

[b31-jhk-41-33] Sirmen B, Atilgan O, Uzun S, Ramazanoglu N, Atil Z, Danismen E (2008). The comparison of static balance and postural sway of water polo players, karate athletes and sedentary people.

[b32-jhk-41-33] Slobounov S, Slobounov E, Newell K (2006). Application of Virtual Reality Graphics in Assessment of Concussion. Cyber Psychology.

[b33-jhk-41-33] Smith AW, Ulmer FF, Wong DP (2012). Gender differences in postural stability among children. J Hum Kinet.

[b34-jhk-41-33] Vuillerme N, Nougier V (2004). Attentional demand for regulating postural sway: The effect of expertise in gymnastics. Brain Res Bull.

[b35-jhk-41-33] Vuillerme N, Nougier V, Prieur JM (2001). Can vision compensate for a lower limbs muscular fatigue for controlling posture in humans?. Neurosci Lett.

[b36-jhk-41-33] Williams AM, Weigelt C, Harris M, Scott MA (2002). Age related differences in vision and proprioception in a lower limb interceptive task: the effects of skill level and practice. Res Q Exerc Sport.

[b37-jhk-41-33] Winter DA (1995). Human balance and posture control during standing and walking. Gait Posture.

[b38-jhk-41-33] Wisemantel S (2002). Talent identification and talent enhancement program in Australian rugby union. Rugby research. A selection of level 3 coaching and refereeing papers.

